# Editorial: Mind the Gap! Criminal justice and health transitions for those with severe mental illness

**DOI:** 10.3389/fpsyt.2023.1143370

**Published:** 2023-02-20

**Authors:** Charlotte Lennox, Beth Angell, Kimberlie Dean

**Affiliations:** ^1^Division of Psychology and Mental Health, University of Manchester, Manchester, United Kingdom; ^2^School of Social Work, University of Michigan, Ann Arbor, MI, United States; ^3^Discipline of Psychiatry and Mental Health, School of Clinical Medicine, University of New South Wales, Sydney, NSW, Australia; ^4^Justice Health and Forensic Mental Health Network, Sydney, NSW, Australia

**Keywords:** criminal justice, health transition, severe mental illness, prison, remand

Care transitions, such as changes in the level of care, the location of care, or the provider of care, are points of vulnerability that can lead to a range of negative outcomes (1–3). Care transitions for those with more complex needs and where care involves multiple agencies can be even more challenging. This is particularly the case for those who may have severe mental illness and who are in contact with the criminal justice system.

The prevalence of mental illness among those in contact with the criminal justice system is much higher than in the general population (4–9). The transition from prison to the community for those with severe mental illness is known to be associated with increased mortality due, predominantly to, suicide and drug overdose, an increased risk of reoffending, poor continuity of health care and treatment pathways, and significant distress (10, 11).

This Research Topic aimed to explore developments or understanding of the issues related to transitional periods for those with severe mental illness in contact with the criminal justice system. By advancing understanding we can inform service developments with the ultimate aim of reducing negative outcomes and preventing people from falling through the gaps.

Tomczak starts our criminal justice transitions journey by focusing on “risky remands.” This article focuses on four individuals who died by suicide/self-inflicted death while on remand in prison. Using underutilized, but publicly available data, the case is made that all too frequently prison is used as a “place of safety” or a pathway into secure healthcare for people with severe mental illness. The cases highlight challenges within police custody with accessing mental health assessment and treatment, as well as missed opportunities to divert those with severe mental illness away from criminal justice pathways.

Fovet et al. present a 10-year retrospective study of psychiatric hospitalizations for people deemed not criminally responsible due to mental illness. The study identifies 3,020 patients meeting this criterion, the majority (88.8%) were males and diagnosed with a psychotic disorder (62%). The majority (87%) were hospitalized in general psychiatric hospitals, with only 13% admitted to maximum-security units. Prior hospitalization was common (73%) as was the rehospitalization rate within 5 years of discharge (62%). The authors found a relatively stable number of people deemed not criminally responsible over the 10 years, which would seem to indicate that people with severe mental illness were not being referred to prison more often than to psychiatric facilities. This again highlights the debate about how best to support those with mental illness in contact with the criminal justice system. As Fovet et al. highlight, there has been a trend toward a “forensification“ (12) of general/community psychiatric services, along with the re-institutionalization of forensic hospitals in many settings (13).

Leonard et al. and D'Orta et al. focus on a particularly vulnerable group, those individuals who revolve within the system e.g., move from prison to mental health units and back to prison. Both studies identified a particular issue for those with a diagnosis of personality disorder. Leonard et al. report that people returned to prison were 4.7 times more likely to have a primary diagnosis of personality disorder, but in contrast were 48% less likely to have a primary diagnosis of Schizophrenia and 69% less likely to have a primary diagnosis of “psychosis other.” D'Orta et al. reported that for revolving door status, the highest odds ratios were found for court-ordered treatments (5.77) and personality disorders (2.14). This is a concern given that many prison settings are inadequately resourced to support this group. In fact, Leonard et al. further found that a fifth of those returned to prison were subsequently placed in segregation and over a fifth had deteriorated to the point of requiring re-referral to hospital.

In the final stage of the criminal justice transition journey Browne et al. and Chowdhury et al. focus on those with severe mental illness in the transition from prison into the community in Australia. Chowdhury et al. found that people with a first diagnosis of psychosis in prison were more likely to have no contact with mental health services post release/discharge than those first diagnosed in hospital with no prior offense record. Browne et al. report that continuity of mental health care for those exiting prison was particularly poor and rates of contact with community mental health teams low in their sample of individuals with severe mental illnesses spending brief periods in custody. These are findings consistent with previous studies (14) and, put together, these findings call for effective multi-agency rehabilitation planning on transition.

There is a small but growing evidence base for interventions to support the success of transition from prison into the community (15, 16), but more evidence is required to fully understand the complexity of the issues. In addition, there are other transitional contexts such as returning to prison from hospital, where currently no evidence-based interventions exist.

## Author contributions

CL supervised the whole process and reviewed submissions. BA and KD reviewed submissions. All authors contributed to the article and approved the submitted version.
